# Antimicrobial resistance and novel mutations detected in the *gyrA* and *parC* genes of *Pseudomonas aeruginosa* strains isolated from companion dogs

**DOI:** 10.1186/s12917-020-02328-0

**Published:** 2020-04-15

**Authors:** Youjin Park, Jaeyoung Oh, Sowon Park, Samuth Sum, Wonkeun Song, Jongchan Chae, Heemyung Park

**Affiliations:** 1 Department of Veterinary Internal Medicine, Konkuk University College of Veterinary Medicine, Seoul, South Korea; 2 Department of Laboratory Medicine, Hallym University College of Medicine, Chuncheon, South Korea; 3 Biotechnology Division, Jeonbuk National University, Iksan, South Korea

**Keywords:** Companion dogs, *P. aeruginosa*, Novel mutations, *gyrA*, *parC*

## Abstract

**Background:**

Fluoroquinolone agents, such as enrofloxacin and marbofloxacin, are commonly used for pseudomonal infection in veterinary medicine. However, the rate of resistance to fluoroquinolones is rapidly increasing, according to multiple studies in various countries. Point mutations in the quinolone resistance-determining region (QRDR) are closely related to the increased fluoroquinolone resistance of *Pseudomonas aeruginosa*. The aim of this study was to investigate current antimicrobial susceptibility and fluoroquinolone resistance in *P. aeruginosa* strains isolated from dogs. The presence of point mutations in the QRDR was confirmed by *gyrA* and *parC* polymerase chain reaction and nucleotide sequencing analysis.

**Results:**

A total of 84 nonduplicated *P. aeruginosa* strains were obtained from 228 healthy dogs (healthy group) and 260 dogs with clinical signs (infected group). Among these isolates, 38 strains from the healthy group were detected in several sample types, whereas 46 strains from the infected group were obtained mostly from dogs’ ears with otitis externa (41/260, 15.8%). All strains were resistant to nalidixic acid, while some were also resistant to enrofloxacin (23/84, 27.4%), marbofloxacin (17/84, 20.2%), levofloxacin (12/84, 14.3%), or ciprofloxacin (11/84, 13.1%). Enrofloxacin resistance was significantly higher in strains from the infected group than in those from the healthy group (*p* < 0.05). Among the 23 fluoroquinolone-resistant strains, 8 and 4 different mutations were detected in the *gyrA* and *parC* genes, respectively. Mutations in *gyrA* were significantly common in the infected group (*p* < 0.05). Hotspots for the *gyrA* and *parC* mutations were Thr83 (34.8%, 8/23) and Pro116 (91.3%, 21/23), respectively. Double and triple mutations were also found in 5 of the strains.

**Conclusion:**

Novel mutations in the *gyrA* and *parC* genes were first found in *P. aeruginosa* isolated from companion dogs in South Korea. These findings suggest that it is important to encourage prudent use of fluoroquinolone antibiotics in canine pseudomonal infection treatment.

## Background

*Pseudomonas aeruginosa* is a gram-negative opportunistic bacterium that infects usually the skin and the urinary and respiratory tracts. In veterinary medicine, fluoroquinolones, such as enrofloxacin and marbofloxacin, are commonly prescribed when a pseudomonal infection is suspected, most commonly for otitis externa/media or urinary tract infections. However, caution should be exerted in the use of antimicrobials to treat pseudomonal infections because of the increasing emergence of antimicrobial-resistant bacteria. The inappropriate use of antimicrobials, increasing prevalence of chronic illness, and lack of environmental barriers between animals and humans has not only contributed to the selection of antimicrobial-resistant bacteria but also imposed a variety of selective pressures that bacteria must face and contend with [1–3].

Quinolones are broad-spectrum antimicrobials that inhibit bacterial DNA gyrase and type IV topoisomerase. In human medicine, the trend of *P. aeruginosa* resistance against quinolones is closely evaluated and monitored. According to the ECDC 2014 surveillance data on antimicrobial resistance and antimicrobial consumption in Europe, the distribution of fluoroquinolone-resistant *P. aeruginosa* is decreasing in many European countries, the United States, and Canada, owing to the development of guidelines for appropriate antimicrobial use by the European Center for Disease Prevention and Control [4]. The fluoroquinolone resistance profile of *P. aeruginosa* strains present in companion dogs has also been evaluated in the United States, France, Brazil, Croatia, and Japan [5–9]. Resistance to fluoroquinolones differs from one country to another or depending on the year the study was performed. The fluoroquinolone resistance profile of *P. aeruginosa* in companion dogs has not been evaluated in South Korea, although enrofloxacin and marbofloxacin are commonly prescribed in veterinary medicine.

*P. aeruginosa* becomes resistant to quinolones through mutation of the quinolone resistance-determining region (QRDR) or plasmid-mediated quinolone resistance (PMQR). A QRDR mutation is a chromosomal point mutation of either DNA gyrase (*gyrA* and *gyrB*) or type IV topoisomerase (*parC* and *parE*), which is the main mechanism of resistance to quinolones in *P. aeruginosa* [6, 10]. A previous study performed in the United States reported that half of the 102 (51.0%) nalidixic acid-resistant *P. aeruginosa* strains isolated from dogs had QRDR mutations [6]. QRDR mutations were also identified in fluoroquinolone-resistant *P. aeruginosa* from dogs in Brazil and Japan [7, 8]. However, the presence of QRDR mutations in *P. aeruginosa* in dogs has not been reported in South Korea. This study was performed to investigate the antimicrobial resistance and mutations in *gyrA* and *parC* in fluoroquinolone-resistant *P. aeruginosa* from companion dogs in South Korea.

## Results

### Distribution of *P. aeruginosa* strains isolated from companion dogs

A total of 84 nonduplicated *P. aeruginosa* strains were isolated from 448 companion dogs either with or without clinical signs from 2017 to 2018. From 228 healthy dogs (healthy group), 38 strains (16.7%) were detected in the ears (14 strains, 6.1%), eyes (10 strains, 4.4%), nasal cavity (7 strains, 3.1%), or rectum (7 strains, 3.1%). However, 46 strains (17.7%) from 260 dogs with clinical signs (infected group) were isolated mostly from the ears (41 strains, 15.8%), and only a few were found in the genitalia (4 strains, 1.5%) or pus (1 strain, 0.4%) (Table 1). Among each specimen type collected from the infected group, *P. aeruginosa* were identified in 1.3% of isolates from genitalia (4/30), 31.5% of isolates from ears (41/130), and 1.3% of isolates from pus (1/8). Therefore, *P. aeruginosa* was isolated mostly from ear samples (55 strains, 11.3%) of companion dogs, which was independent of their health status.

**Table 1:** Distribution of nonduplicated *Pseudomonas aeruginosa* from healthy and infected dogs based on specimen types

Specimens	No. (%) isolates		
	Healthy group (*n* = 228)	Infected group ^*a*^	
		Total (*n* = 260)	Specimen type
Eye	10 (4.4)	0 (0.0)	0/2 (0.0)
Rectum	7 (3.1)	0 (0.0)	0/10 (0.0)
Genitalia	–	4 (1.5)	4/30 (1.3)
Ear	14 (6.1)	41 (15.8)	41/130 (31.5)
Nasal cavity	7 (3.1)	0 (0.0)	0/80 (0.0)
Pus	–	1 (0.4)	1/8 (1.3)
Total	38 (16.7)	46 (17.7)	

^*a*^ Disease name of the infected group: genitalia, pyometra; ear, otitis externa; pus, bronchitis

### Antimicrobial resistance profile

**Fig. 1:**
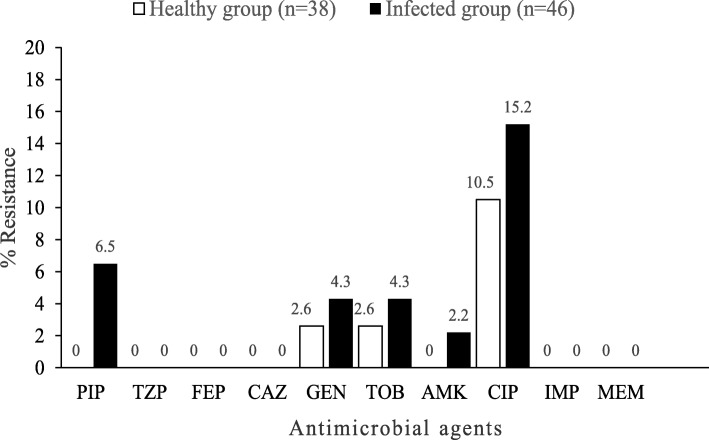
Comparison of antimicrobial resistance of *Pseudomonas aeruginosa* from healthy and infected dogs. PIP, piperacillin; TZP, piperacillin-tazobactam; FEP, cefepime; CAZ, ceftazidime; GEN, gentamicin; TOB, tobramycin; AMK, amikacin; CIP, ciprofloxacin; IMP, imipenem; MEM, meropenem

Strains were tested for resistance to 10 different antibiotics (Table 2*, *Fig. 1). In the healthy group, resistance to ciprofloxacin was the most frequent (n = 4, 10.5%), and resistance to ciprofloxacin-gentamicin-tobramycin, gentamicin, and tobramycin was observed for one organism. In the infected group, resistance to ciprofloxacin was the most frequent (n = 7, 15.2%), followed by that to piperacillin (n = 3, 6.5%), ciprofloxacin-gentamicin-tobramycin (n = 2, 4.3%), gentamicin (n = 2, 4.3%), tobramycin (n = 2, 4.3%) and amikacin (n = 1, 2.2%). No strain was resistant to cefepime, ceftazidime, colistin, imipenem, meropenem, or piperacillin-tazobactam. Additionally, resistance to piperacillin and amikacin was observed only in strains from the infected group. While resistance to ciprofloxacin was apparently higher in the infected group than in the healthy group, there was no significant difference in the frequency of resistance to ciprofloxacin between the healthy and infected groups (*p*-value 0.747).

**Table 2:** Antimicrobial resistance patterns of *Pseudomonas aeruginosa* from healthy and infected dogs

Antimicrobial resistance pattern ^*a*^	No. (%) resistant strains		
	Healthy group (*n* = 38)	Infected group (*n* = 46)	Total (*n* = 84)
PIP	0 (0.0)	3 (6.5)	3 (3.6)
CIP	4 (10.5)	7 (15.2)	11 (13.1)
CIP-GEN-TOB	1 (2.6)	2 (4.3)	3 (3.6)
AMK	0 (0.0)	1 (2.2)	1 (1.2)
GEN	1 (2.6)	2 (4.3)	3 (3.6)
TOB	1 (2.6)	2 (4.3)	3 (3.6)

^*a*^ PIP, piperacillin (zone diameter resistance breakpoint, ≤14 mm); GEN, gentamicin (zone diameter resistance breakpoint, ≤12 mm); TOB, tobramycin (zone diameter resistance breakpoint, ≤12 mm); AMK, amikacin (zone diameter resistance breakpoint, ≤14 mm); CIP, ciprofloxacin (zone diameter resistance breakpoint, ≤15 mm)

The MICs for 4 fluoroquinolone antibiotics, including nalidixic acid, were evaluated (Table 3). All *P. aeruginosa* strains from both groups were resistant to nalidixic acid (MIC ranges: 64 to ≥256 μg/mL in the healthy group and 32 to ≥256 μg/mL in the infected group). The highest percentage of resistance among the 4 fluoroquinolone agents was seen for enrofloxacin (breakpoint, ≥ 4 μg/mL), with 15.8% (6/38) resistant strains from the healthy group and 37.0% (17/46) from the infected group. The percentage of resistance to marbofloxacin (≥ 4 μg/mL) was 13.2% (5/38) for strains from the healthy group and 26.1% (12/46) for those from the infected group, and resistance (≥ 8 μg/mL) to levofloxacin was 13.2% (5/38) for the healthy group strains and 15.2% (7/46) for those from the infected group. Moreover, resistance (≥ 4 μg/mL) to ciprofloxacin was observed for 10.5% (4/38) of strains from the healthy group and 15.2% (7/46) of strains from the infected group (Table 3). Collectively, strains from the infected group were significantly more resistant to enrofloxacin and less susceptible to marbofloxacin than those from the healthy group (*p* < 0.05).

**Table 3:** Distribution of fluoroquinolone-resistant *Pseudomonas aeruginosa* from healthy and infected dogs

Antimicrobial agents	MIC (μg/mL)		No. (%) resistant strains		*p*-value
	Range	Breakpoint	Healthy group (*n* = 38)	Infected group (*n* = 46)	
Nalidixic acid	0.05–256	≥32	38 (100.0)	46 (100.0)	n/a
Ciprofloxacin	0.015–32	≥4	4 (10.5)	7 (15.2)	0.747
Levofloxacin	0.015–32	≥8	5 (13.2)	7 (15.2)	0.700
Enrofloxacin	0.03–16	≥4	6 (15.8)	17 (35.4)	0.031 ^*a*^
Marbofloxacin	0.03–16	≥4	5 (13.2)	12 (26.1)	0.142

^*a*^ Statistically significant (*p* < 0.05)

### Amino acid variations in the *gyrA* and *parC* gene products

Given that strains resistant to enrofloxacin among fluoroquinolone antibiotics were the most abundant, sequencing analysis of the *gyrA* and *parC* genes from the 23 enrofloxacin-resistant *P. aeruginosa* strains was performed (Table 4). QRDR mutations were identified in 6 (15.8%) out of 38 strains from the healthy group and in 17 (37%) out of 46 strains from the infected group; all 23 mutations belonged to the 23 enrofloxacin-resistant strains of *P. aeruginosa*. In addition, 11 (47.8%) of these 23 strains were resistant to ciprofloxacin. Of the amino acid substitutions found in the 23 fluoroquinolone-resistant strains, 8 were present in the *gyrA* gene and 4 in the *parC* gene. Regarding *gyrA*, the mutation Thr83Ile was found in 2 strains from the healthy group and in 4 strains from the infected group; in addition, other mutations were found in strains from the infected group: Asp87Gly in 2 strains, Thr83Ile-Asp87Gly in 1 strain, Leu55Gln-Asp82Asn-Thr83Ala in 1 strain, and Asp87Asn in 1 strain. Notably, the novel triple-nucleotide mutation found in *gyrA*, leading to codon changes Leu55 → Gln, Asp82 → Asn, and Thr83 → Ala, corresponds to a *P. aeruginosa* strain from the infected group with high MIC values for the four fluoroquinolone agents tested. Moreover, the strain with two nucleotide mutations, i.e., Thr83Ile and Asp87Gly, showed identical MIC values for these four antibiotics. Mutations in *gyrA* were significantly more common in strains from the infected group than in those from the healthy group (*p* < 0.05).

**Table 4:** Comparison of quinolone resistance-determining regions among fluoroquinolone-resistant *Pseudomonas aeruginosa* between healthy and infected dogs

Strain no.	Amino acid substitution ^*a*^		MIC (μg/mL) ^*b*^			
	*gyrA*	*parC*	CIP	LVX	ENR	MFX
***Healthy group***						
PAE18	–	Pro116Arg	4	16	≥16	≥16
PAE43	Thr83Ile	Pro116Arg	2	8	≥16	8
PAE51	–	Pro116Arg	16	16	≥16	≥16
PAE52	Thr83Ile	Ser87Leu, Pro116Arg	≥32	≥32	≥16	≥16
PAE71	–	Pro116Arg	≥32	≥32	≥16	≥16
PAE85	–	Pro116Arg	0.25	0.5	4	1
***Infected group***						
KVNON14	Asp87Gly	Pro116Arg	4	8	≥16	≥16
KVNON23	Thr83Ile	Pro116Arg	≥32	≥32	≥16	≥16
KVNON33	Thr83Ile, Asp87Gly	Ser87Leu, Pro116Arg	≥32	≥32	≥16	≥16
KVNON47	–	Pro116fs	0.5	2	4	2
KVNON66	Asp87Gly	Pro116Arg	4	8	≥16	≥16
KVNON127	Asp87Tyr	Pro116Arg	1	2	4	4
KVNON184	–	Pro116Ala	0.125	0.5	8	1
KVNON194	Leu55Gln, Asp82Asn, Thr83Ala	Ser87Leu, Pro116Ala	≥32	≥32	≥16	≥16
KVNON199	Thr83Ile	Pro116Arg	8	32	≥16	≥16
KVNON216	–	Pro116Arg	0.5	2	4	2
KVNON219	Thr83Ile	Pro116Arg	1	4	8	8
KVNON271	–	Pro116Arg	2	4	4	2
KVNON279	Thr83Ile	Pro116Arg	4	8	≥16	≥16
KVNON324	–	Pro116Arg	0.5	4	4	4
KVNON442	Asp87Asn	Pro116Arg	0.5	2	4	4
KVNON508	–	Pro116fs	0.5	2	4	2
KVNON509	–	Pro116Arg	2	2	4	4

^*a*^ Amino acid substitutions in *gyrA* and *par*C: Thr83Ile, ACC → A**T**C; Thr83Ala, ACC → **G**CC; Asp87Gly, GAC → G**G**C; Leu55Gln, CTG → C**A**G; Asp82Asn, GAC → **A**AC; Asp87Asn, GAC → **A**AC; Asp87Tyr, GAC → **T**AC; Pro116Arg, CCG → C**G**G; Pro116Ala, CCG → **G**CG; Ser87Leu, TGC → T**T**G; Pro116fs, CGG → CG-. Ala, alanine; Arg, arginine; Asn, asparagine; Asp, aspartic acid; Cys, cysteine; Gln, glutamine; Gly, glycine; Ile, Isoleucine; Leu, leucine; Pro, proline; Ser, serine; Thr, threonine; Tyr, tyrosine; fs, frameshift

^*b*^*NAL* nalidixic acid, *CIP* ciprofloxacin, *LVX* levofloxacin, *ENR* enrofloxacin, *MFX* marbofloxacin

Regarding the *parC* gene, mutations were observed in all 23 enrofloxacin-resistant *P. aeruginosa* strains. A novel single nucleotide mutation at *parC* codon 116, resulting in a Pro116 → Arg mutation, was found in 21 (91.3%) of the strains, which included a double nucleotide alteration at codons 87 and 116, Ser87 → Leu and Pro116 → Arg, that was observed in 1 strain from the healthy group and in 2 strains from the infected group. In addition, a frameshift (fs) mutation due to a nucleotide deletion at *parC* codon 116, Pro116fs, CGG → CG-, was detected in 2 strains from the infected group. Therefore, mutation hotspots found for *gyrA* and *parC* were Thr83Ile (*n* = 7) and Pro116Arg (*n* = 21), respectively. There was no significant difference between the number of point mutations in either the *gyrA* or the *parC* genes and the MIC values for fluoroquinolone antimicrobial agents among resistant strains. In addition, QRDR mutations in the *gyrA* or *parC* genes conferring intermediate resistance to enrofloxacin (MIC: 1 to 2 μg/mL) were not found in the *P. aeruginosa* strains.

## Discussion

*P. aeruginosa* often causes otitis externa/media and cystitis in companion dogs. Despite the regular usage of fluoroquinolone agents, such as enrofloxacin and marbofloxacin, in the treatment of infected dogs, neither the antimicrobial susceptibility nor the quinolone-resistance profile of *P. aeruginosa* strains has ever been studied in South Korea. This study aimed to reveal the antimicrobial susceptibility and quinolone resistance of *P. aeruginosa* strains isolated from both healthy and clinically ill companion dogs. *P. aeruginosa* was recovered from several samples in dogs in the healthy group; nevertheless, one-third of these strains were obtained from ears. In addition, *P. aeruginosa* was isolated mainly from the ears of dogs in the infected group. These results are consistent with *P. aeruginosa* as a common agent causing otitis. The fact that one-third of the strains from the healthy group were obtained from ears suggests a history of otitis externa/media in dogs that appear to be healthy.

*P. aeruginosa* strains from this study were not resistant to a variety of antimicrobials, as tested by disk diffusion assays, except for ciprofloxacin, gentamicin, tobramycin, and amikacin. The resistance to these latter antimicrobial agents may be due to the prevalence of antimicrobial use for treating diseases in animal hospitals. It is important to note that a significant difference in the resistance frequencies to ciprofloxacin between the healthy and infected groups was not found. These results could be explained if the usage of ciprofloxacin, among quinolone antibiotics, is less common in animal hospitals for treating diseases; then, it would be possible to hypothesize that the companion dogs in this study had not been exposed to ciprofloxacin.

MIC values for fluoroquinolones were different among antibiotics. All strains showed high resistance rate to enrofloxacin but low resistance rate to ciprofloxacin. These results are similar to *P. aeruginosa* resistance trends reported in other countries. Previous resistance reports indicate that 13.1% of strains from this study, 16% from the United States, 13% from Spain, 2.2% from Croatia, 4.8% from Brazil, and 20% from Japan are resistant to ciprofloxacin [6–9]. Moreover, the reported percentages of resistance to enrofloxacin and marbofloxacin were 27.4 and 20.2% in this study, 31 and 27% in the United States, 21.7 and 9% in Spain, and 15.6 and 8.9% in Brazil, respectively, whereas 31.5% resistance to enrofloxacin was reported in Japan. According to a previous study, *P. aeruginosa* strains show their highest resistance to enrofloxacin, the most commonly used fluoroquinolone in veterinary medicine [6]. In Croatia, resistance to enrofloxacin and marbofloxacin significantly increased from 2.7 to 15.6% and from 4.4 to 8.9%, respectively, since the use of marbofloxacin, in addition to enrofloxacin, in veterinary medicine was approved in the country [9, 11]. Our results show that resistance to marbofloxacin was 20.2%, which is higher than that reported for strains from Spain and Croatia [8, 9]. This finding may be related to differences in the frequency of marbofloxacin prescription in each country. Since the usage of marbofloxacin for treating clinically ill dogs was approved in South Korea, follow-up studies should be undertaken to determine the evolution of fluoroquinolone-resistant *P. aeruginosa* strains.

Previous studies have also reported different susceptibilities or resistances to enrofloxacin depending on the origin of the studied isolates; ear isolates, for example, show a significantly higher resistance to enrofloxacin than skin isolates [12]. Other studies have reported increased resistance rates in ear isolates [6, 13]. In Croatia, resistance to enrofloxacin increased from 1.0% in 2011 to 8.9% in 2017, perhaps because Mekić et al. evaluated only the resistance of isolates from dogs with otitis externa [9, 14]. *P. aeruginosa* strains isolated from ears in this study showed a high susceptibility to ciprofloxacin (82.5%) and a low susceptibility to both enrofloxacin (17.5%) and marbofloxacin (3.5%). This trend is similar to that reported in Croatia, where the use of marbofloxacin has been licensed only for treating otitis externa. As mentioned, in this study, strains from ears were significantly less susceptible to marbofloxacin than isolates from other body parts (*p* < 0.05). This finding suggests that caution should be taken in the usage of enrofloxacin or marbofloxacin in dogs with otitis to avoid an increase in rates of resistance to these antibiotics.

In the present study, novel QRDR mutations in both the *gyrA* and *parC* genes were detected in fluoroquinolone-resistant *P. aeruginosa*. Among QRDR genes (*gyrA*, *gyrB*, *parC*, and *parE*), QRDR mutations are most frequently detected in *gyrA* and *parC* [6–8]. In this study, the same point mutation within *parC* was found in all enrofloxacin-resistant strains, whereas point mutations in *gyrA* were almost exclusively detected in strains from the infected group. The most commonly reported point mutations or hotspots are Thr83 and Asp87 for *gyrA* and Ser87 for *parC* in both dogs and humans [6, 8, 15]. Consistently, the hotspot for *gyrA* was Thr83 (*n* = 8, 38.1%) in this study. In contrast, a point mutation at Pro116 of *parC* (*n* = 21, 80.8%) in this study was the most frequently observed mutation found in strains from this study. This sequence may be a wild-type allele of canine *P. aeruginosa* strains from South Korea rather than a QRDR mutation resulting in increased resistance to fluoroquinolones. Nevertheless, a high correlation between QRDR mutations and increased MICs to fluoroquinolones has been reported previously [7]. In this study, the MIC values for the four fluoroquinolone antibiotics studied were not significantly different for strains with different *gyrA* mutations, while *parC* mutations were detected in all 23 enrofloxacin-resistant strains. As a result, the novel Pro116 alteration described in *parC*, which has never been reported in nalidixic acid-resistant gram-negative bacteria until now, is suspected to be correlated with increased enrofloxacin resistance.

## Conclusions

The high resistance to enrofloxacin and the occurrence of QRDR mutations in *P. aeruginosa* strains from clinically ill dogs may reflect the increasing use of enrofloxacin in animal hospitals. However, further investigation is warranted regarding the pattern and frequency of antimicrobial use in dogs. Overall, this study provides a warning about the inappropriate use of fluoroquinolone agents in the treatment of pseudomonal infections in companion dogs.

## Methods

### Sample collection

To investigate the antimicrobial susceptibility and fluoroquinolone resistance mechanisms of *P. aeruginosa* strains isolated from companion dogs with or without present clinical signs, sampling was performed on a total of 50 veterinary hospitals from five Korean provinces between 2017 and 2018. Samples were collected from the ears, nasal cavity, eyes, and rectum of dogs that were clinically healthy and had not taken any antibiotics within the last six months, using sterile transport swabs (COPAN, Brescia, Italy). Using the same sampling method, samples from the ears, nasal cavity, eyes, genitalia, rectum, and pus were collected from dogs with clinical signs of bacterial infection. Isolates were first classified according to their origin as belonging either to the healthy group or to the infected group. All samples collected from companion animals were collected under the owner’s approval. Every individual was provided with information regarding the purpose and method of sampling. The need for ethics approval was deemed unnecessary according to the national regulations.

### Isolation and identification

Swab samples were cultured on trypticase soy broth (TSB, Becton, Dickinson and Company, Sparks, MD, USA) for 18 h at 37 °C. Then, they were streaked on MacConkey agar (Becton, Dickinson and Company) and incubated for 18 h at 37 °C. Those isolates that were identified as *P. aeruginosa* by Gram staining (Gram stain set, Remel, Dartfold, UK), oxidase tests (Becton, Dickinson and Company), and a microbial identification system (VITEK® MS, bioMérieux, Marcy-l’Étoile, France) were selected for further analysis.

### Determination of antimicrobial susceptibility and minimum inhibitory concentrations

Antimicrobial susceptibility analyses were performed by the disk diffusion test, according to the Clinical Laboratory of Standard Institute guidelines [16]. Eleven different antimicrobial disks were used (Oxoid Limited, Basingstoke, UK) for testing: piperacillin, piperacillin-tazobactam, cefepime, ceftazidime, ciprofloxacin, gentamicin, amikacin, tobramycin, meropenem, and imipenem.

The minimum inhibitory concentrations (MICs) for fluoroquinolone antimicrobials were determined as indicated by the CLSI broth microdilution method. The quinolones and fluoroquinolones used were nalidixic acid (Sigma-Aldrich, Germany), ciprofloxacin (Sigma-Aldrich), levofloxacin (Sigma-Aldrich), enrofloxacin (Bayer Vital GmbH, Leverkusen, Germany), and marbofloxacin (Vetoquinol, Lure, France). *P. aeruginosa* ATCC 27853 and *Escherichia coli* ATCC 25922 were used as quality control strains. The breakpoints for ciprofloxacin (≥4 μg/mL) and levofloxacin (≥8 μg/mL) were followed based on CLSI guidelines, whereas the breakpoints for nalidixic acid (≥32 μg/mL) was followed as described by Rubin et al., 2008 and for enrofloxacin (≥4 μg/mL) and marbofloxacin (≥4 μg/mL) as described by Pintarić et al., 2017.

### PCR amplification and sequencing analysis

Genomic DNA was extracted from all *P. aeruginosa* strains using a QIAamp DNA Mini kit (QIAGEN, Hilden, Germany) according to the manufacturer’s instructions. PCR amplification of the *gyrA* and *parC* genes was performed as previously described (Rubin et al., 2008). Purified PCR products were sequenced using an automated 3730xl DNA Analyzer (Applied Biosystems, Foster City, CA, USA), and the resulting sequences were compared against the complete genome sequence of *P. aeruginosa* ATCC 27853 (GenBank accession number, CP015117) using both the BLAST network service and the ClustalW multiple sequence alignment program (www.genome.jp/tools-bin/clustalw).

### Statistical analysis

The obtained data are presented as the relative frequency from the values. A *t*-test and Pearson’s Chi-square test were used to compare the antimicrobial susceptibility profiles between the healthy group and the infected group. All statistical analyses were performed by using SPSS 20.0 software (IBM SPSS, Inc., Chicago, IL, USA). The results were considered statistically significant for *p* values less than 0.05.

### Nucleotide sequence accession numbers

Newly described sequences for the gyrA and parC genes have been assigned the following GenBank accession numbers: MN068218 to MN068220 (*parC* mutations) and MN068221 (*gyrA* mutation).

## Abbreviations

Ala: Alanine
AMK: Amikacin
Arg: Arginine
Asn: Asparagine
Asp: Aspartic acid
CIP: Ciprofloxacin
CLSI: Clinical and Laboratory Standards Institute
Cys: Cysteine
ECDC: European Centre for Disease Prevention and Control
ENR: Enrofloxacin
Fs: Frameshift
GEN: Gentamicin
Gln: Glutamine
Gly: Glycine
Ile: Isoleucine
Leu: Leucine
LVX: Levofloxacin
MFX: Marbofloxacin
MIC: Minimum inhibitory concentration
NAL: Nalidixic acid
PCR: Polymerase chain reaction
PIP: Piperacillin
Pro: Proline
QRDR: Quinolone resistance-determining region
Ser: Serine
Thr: Threonine
TOB: Tobramycin
Tyr: Tyrosine
